# LIN9, a Subunit of the DREAM Complex, Regulates Mitotic Gene Expression and Proliferation of Embryonic Stem Cells

**DOI:** 10.1371/journal.pone.0062882

**Published:** 2013-05-07

**Authors:** Jasmina Esterlechner, Nina Reichert, Fabian Iltzsche, Michael Krause, Florian Finkernagel, Stefan Gaubatz

**Affiliations:** 1 Theodor Boveri Institute, Biocenter, University of Wuerzburg, Wuerzburg, Germany; 2 Institute for Molecular Biology and Tumor Research (IMT), University of Marburg, Marburg, Germany; Baylor College of Medicine, United States of America

## Abstract

The DREAM complex plays an important role in regulation of gene expression during the cell cycle. We have previously shown that the DREAM subunit LIN9 is required for early embryonic development and for the maintenance of the inner cell mass *in vitro*. In this study we examined the effect of knocking down LIN9 on ESCs. We demonstrate that depletion of LIN9 alters the cell cycle distribution of ESCs and results in an accumulation of cells in G2 and M and in an increase of polyploid cells. Genome-wide expression studies showed that the depletion of LIN9 results in downregulation of mitotic genes and in upregulation of differentiation-specific genes. ChIP-on chip experiments showed that mitotic genes are direct targets of LIN9 while lineage specific markers are regulated indirectly. Importantly, depletion of LIN9 does not alter the expression of pluripotency markers SOX2, OCT4 and Nanog and LIN9 depleted ESCs retain alkaline phosphatase activity. We conclude that LIN9 is essential for proliferation and genome stability of ESCs by activating genes with important functions in mitosis and cytokinesis.

## Introduction

Pluripotent embryonic stem cells (ESCs) are derived from the inner cell mass (ICM) of the pre-implantation embryo. ESCs have the unique ability to self-renew while retaining the ability to differentiate into any cell type of the adult animal. Self-renewal and pluripotency of ESCs is maintained by a set of pluripotency transcription factors such as SOX2, OCT4 and Nanog and by polycomb group complexes that maintain the undifferentiated state of ESCs through the repression of developmental genes [1]. Inactivation of pluripotency factors or polycomb proteins leads to loss of pluripotent cells and aberrant differentiation. Embryonic stem cells are also characterized by an unusual cell cycle. ESCs proliferate much faster than differentiated cells due to very short G1 and G2 phases [2], [3]. The abnormal cell cycle structure of ESCs is intimately linked to the unique features of ESCs. However, the relationship between cell cycle regulation and ESC pluripotency is incompletely understood. For example, while it has been suggested that the short G1 phase of ESCs inhibits their differentiation and preserves pluripotency, a recent study showed that a short G1 phase is not sufficient to prevent differentiation of ESCs [4].

LIN9 is a subunit of the evolutionary conserved DREAM complex that was first described in Drosophila and was shown to function as a repressor of cell cycle regulated genes [5], [6]. In human cells DREAM consists of a five-protein core module and associated proteins [7], [8]. DREAM undergoes a cell cycle dependent switch of subunits. Specifically, in quiescent cells the DREAM core module binds to the E2F4 transcription factor and to the p130 retinoblastoma protein to establish a repressor complex. In the S-phase, binding to E2F4/p130 is lost and DREAM now associates with the transcription factor B-MYB, yielding a transcriptional activator complex. DREAM-B-MYB has a crucial role in cell cycle progression. Specifically, it is required for normal progression through mitosis and cytokinesis because it activates a set of key mitotic genes such as Cyclin B, BUB1 and Aurora A [9], [7], [8], [10]. DREAM is also essential during early embryonic development, since mice lacking the DREAM subunit LIN9 or B-MYB die shortly after implantation because the inner cells mass (ICM) is not maintained after implantation [11], [12]. B-MYB is also required in ESCs for proper expression of critical cell cycle regulators such as Cyclin B1 and PLK1 and pluripotency genes such as OCT4 and SOX2 [13], [14]. Thus, B-MYB is important not only for cell cycle progression but also for the pluripotent state of ESCs and both functions may contribute to the early lethal phenotype of B-MYB mutant embryos. Given that LIN9 and B-MYB are both subunits of the DREAM complex together with the finding that they are essential at a similar stage during embryonic development, it is possible that DREAM also plays important roles in ESC proliferation and pluripotency. However the role of the DREAM core complex in ESCs has not been investigated.

To explore the function of DREAM in ESCs, we depleted the conserved core-subunit LIN9 by RNAi and found that it is required for normal cell cycle progression and genome stability. Microarray and ChIP-on-chip experiments showed that mitotic genes are direct targets of LIN9 in ESCs. Although depletion of LIN9 also resulted in upregulation of some lineage specific genes, the cells remained undifferentiated and the expression of key stem cell regulators was maintained. Together our results indicate a role for LIN9 in gene expression at mitosis in ESCs.

## Results

### Lin9 Expression in Mouse Pre-implantation Embryos

**Figure 1:**
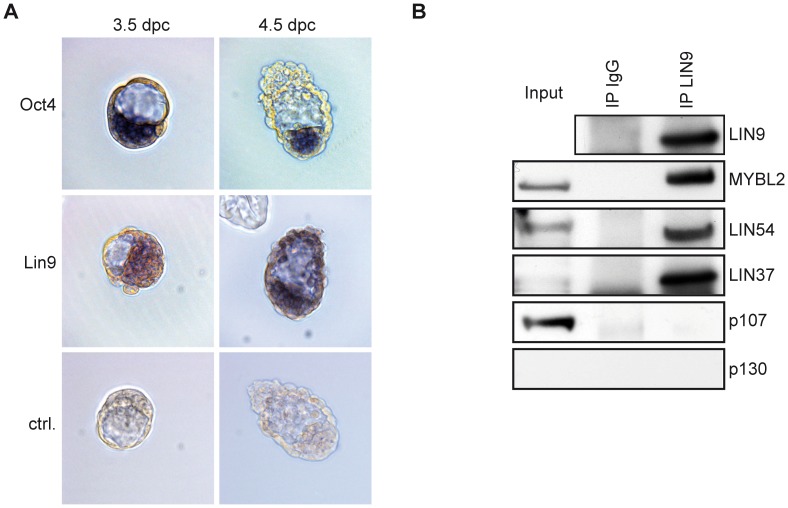
Expression of Lin9 in pre-implantation embryos and ESCs. (A) Expression of *Lin9* mRNA in 3.5 and 4.5 dpc blastocysts was analyzed by *in situ* hybridization with a DIG-labeled Lin9 probe. As a control, Oct4 mRNA was analyzed. (B) Nuclear lysates from ESCs were immunoprecipitated with a LIN9 antibody or with nonspecific IgG as a control. Co-precipitated proteins were detected with the antibodies indicated. Input (5%) was also loaded on the gel for comparison.

We have previously shown that deletion of *Lin9* in mice leads to early embryonic lethality at the peri-implantation stage [11]. LIN9 is also required for maintenance of the inner cell mass (ICM) *in vitro* in pre-implantation embryos [11]. To further analyze the reason for embryonic lethality, we investigated the expression of *Lin9* mRNA in 3.5 and 4.5 dpc blastocysts by *in situ* hybridization. *Oct4*, an epiblast marker, was used as a control [15]. *Lin9* mRNA was detected in the ICM of 3.5 and 4.5 embryos (Fig. 1A). In addition it could also be detected in the trophoectoderm of 4.5 embryos. *Oct4* showed ICM specific expression, as expected (Fig. 1A). The expression of *Lin9* in pre-implantation embryos is consistent with its role in early development.

### LIN9 is a Component of the DREAM Complex in ESCs

We next investigated the expression of LIN9 in ESCs, which are derived from the inner cell mass of pre-implantation blastocysts [16]. LIN9 was immunoprecipitated from nuclear lysates. By immunoblotting with an antibody directed at LIN9, a single band was detected at the expected size, confirming that the LIN9 protein is expressed in ESCs (Fig. 1B). B-MYB, LIN54 and LIN37 co-precipitated with LIN9, indicating that LIN9 is part of the DREAM complex in ESCs (Fig. 1B). Interactions with DREAM subunits were specific, because no signal was observed with immunprecipitations with nonspecific IgG. The LIN52 subunit of DREAM and RbAp48 could not be analyzed because of the lack of suitable antibodies directed at the mouse proteins. p130 was not expressed in ESCs and therefore no interaction with LIN9 was detected (Fig. 1B). Although p107 is expressed in growing ESCs, it did not co-precipitate with LIN9. Thus, LIN9 associates with the core components of DREAM and with B-MYB in ESCs but not with pocket proteins. In summary, LIN9 is expressed in pre-implantation embryos and in ESCs and it could play a role in cell cycle regulation or differentiation of ESCs.

### Impaired Embryoid Body Formation Upon Depletion of LIN9 in ESCs

**Figure 2:**
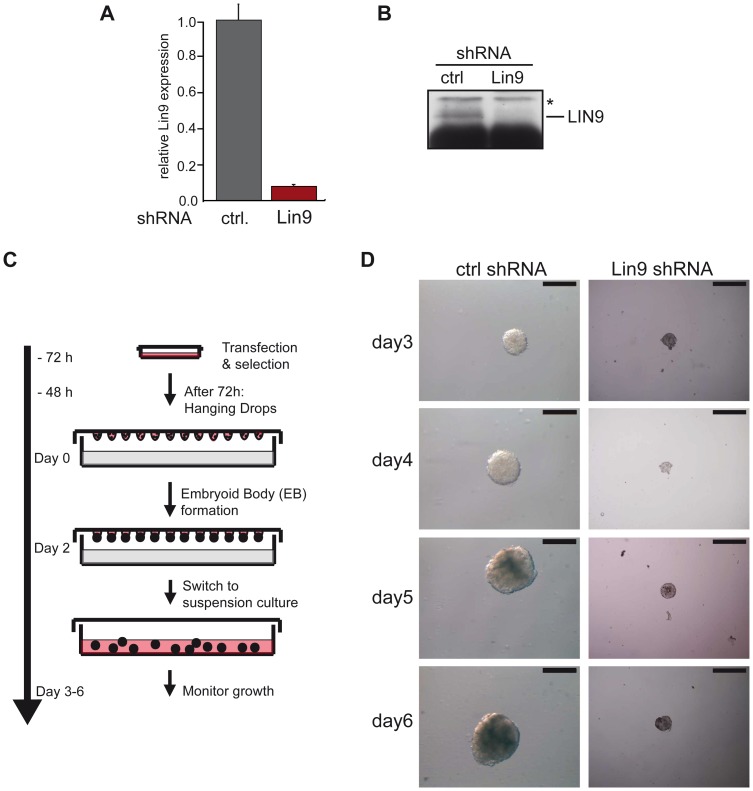
Impaired embryoid body formation after depletion of LIN9 in ESCs. ESCs were transfected with a plasmid encoding a LIN9 specific shRNA. LIN9 mRNA (A) and protein levels (B) were compared with the levels in control-transfected cells by RT-qPCR and immunoblotting. (C) Embryoid body formation: Outline of the experiment. Equal numbers of LIN9 depleted ESCs or control cells were placed in hanging drops on lids of cell culture dishes. After two days, embryoid bodies were harvested and grown in suspension in the absence of LIF for up to 6 days. (D) Embryoid bodies formed in control cells and LIN9-depleted cells. Scale bar: 100 µM. See Figure S1 for additional examples of embryoid bodies formed in control cell and LIN9 depleted cells from an independent experiment.

Because LIN9 is required for early embryonic development, we next wanted to analyze the developmental capacity of LIN9 depleted ESCs. The formation of embryoid bodies is an accepted *in vitro* model of differentiation and development [17]. Embryoid body formation can be triggered by culturing the ESCs under nonadherent conditions in the absence of leukemia inhibitory factor (LIF). To investigate the role of LIN9 in embryoid body formation, we transfected ESCs with a vector encoding a puromycin resistance gene and a LIN9-specific shRNA. After 3 days of puromycin selection, the efficiency of LIN9 depletion was tested at the mRNA and protein level. A significant decrease in LIN9 mRNA and protein was detected by RT-qPCR and immunoblotting in cells expressing the LIN9-specific shRNA compared to a control shRNA (Fig. 2A,B ). Next, equal number of control cells and LIN9 depleted cells were seeded in hanging drops for embryoid body formation (Fig. 2C). Two days later, embryoid bodies were harvested and plated onto dishes coated with poly-hema in the absence of LIF. Control transfected ESCs increased in size during the course of the experiment (Fig. 2D, Supplemental Fig. S1). In contrast, embryoid bodies in LIN9 depleted cells were much smaller compared to controls and did not significantly increase in size when cultured for up to 6 days (Fig. 2D).

### Depletion of LIN9 in ESCs by RNAi Results in Accumulation of Cells with 4n DNA Content and of Polyploid Cells

**Figure 3:**
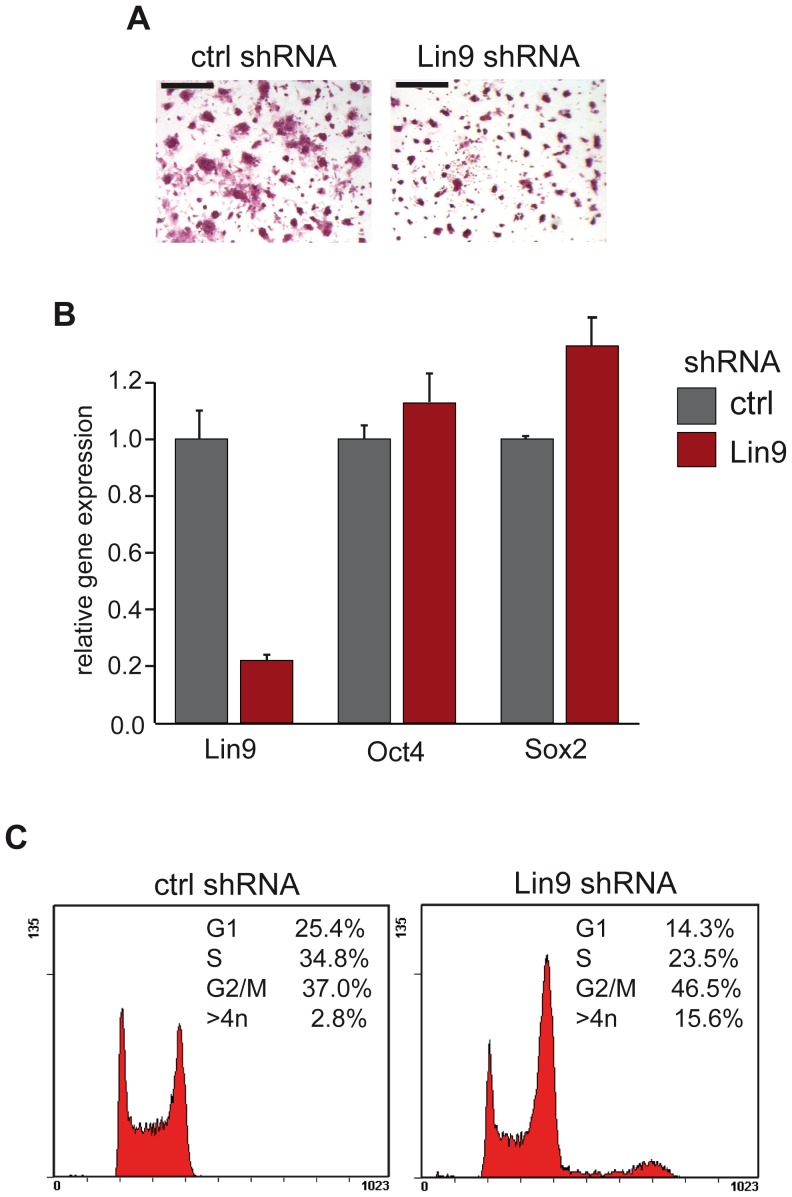
Cell cycle arrest in G2/M after depletion of LIN9. (A) Alkaline-phosphatase (AP) staining of control cells and LIN9 depleted cells. Scale bar: 200 µM (B) Expression of pluripotency markers Oct4 and Sox2 was analyzed in control-depleted cells and LIN9 depleted cells by RT-qPCR. (C) The cell cycle profile of LIN9 depleted ESCs and of control cells was analyzed by flow cytometry.

To investigate whether the reduced size of embryoid bodies is due to changes in the differentiation status of the ESCs, we performed alkaline phosphatase (AP) staining. AP activity can be detected only in undifferentiated ESCs and is rapidly lost when the cells differentiate. Therefore the AP assay can be used to assess the self-renewal capacity of ESCs. AP staining was performed with cells transfected with the LIN9 specific shRNA or with a control shRNA. LIN9 depleted cells maintained alkaline phosphatase activity indicating that the cells remain undifferentiated (Fig. 3A). However, LIN9-deficient colonies were smaller and contained fewer cells than control colonies. Pluripotent ESCs are maintained in an undifferentiated state by a set of key transcription factors including Oct4 and Sox2. The expression levels of Oct4 and Sox2 did not differ between control cells and LIN9-depleted cells, indicating that LIN9 is not required for maintaining the expression of these pluripotency genes. These data are consistent with the maintenance of AP activity in LIN9 depleted cells (Fig. 3B).

The reduction in cell number could be due to changes in cell cycle progression. To investigate whether depletion of LIN9 leads to changes in cell cycle progression, we compared the cell cycle profiles of LIN9-depleted cells with that of control cells by flow cytometry. After depletion of LIN9 for 3 days, a significant decrease in the proportion of cells in G1 and S-phase and an increase in the proportion of cells in G2/M was observed (Fig. 3C). Of note, polyploid cells with >4n DNA content were also significantly increased from 2.8% in control cells to 15.6% in LIN9 depleted cells. These results indicate that LIN9 is required for normal cell cycle progression and for genome stability of ESCs.

### Knockdown of LIN9 Results in the Reduction of Mitotic Gene Expression and in Induction of Differentiation Markers

**Figure 4:**
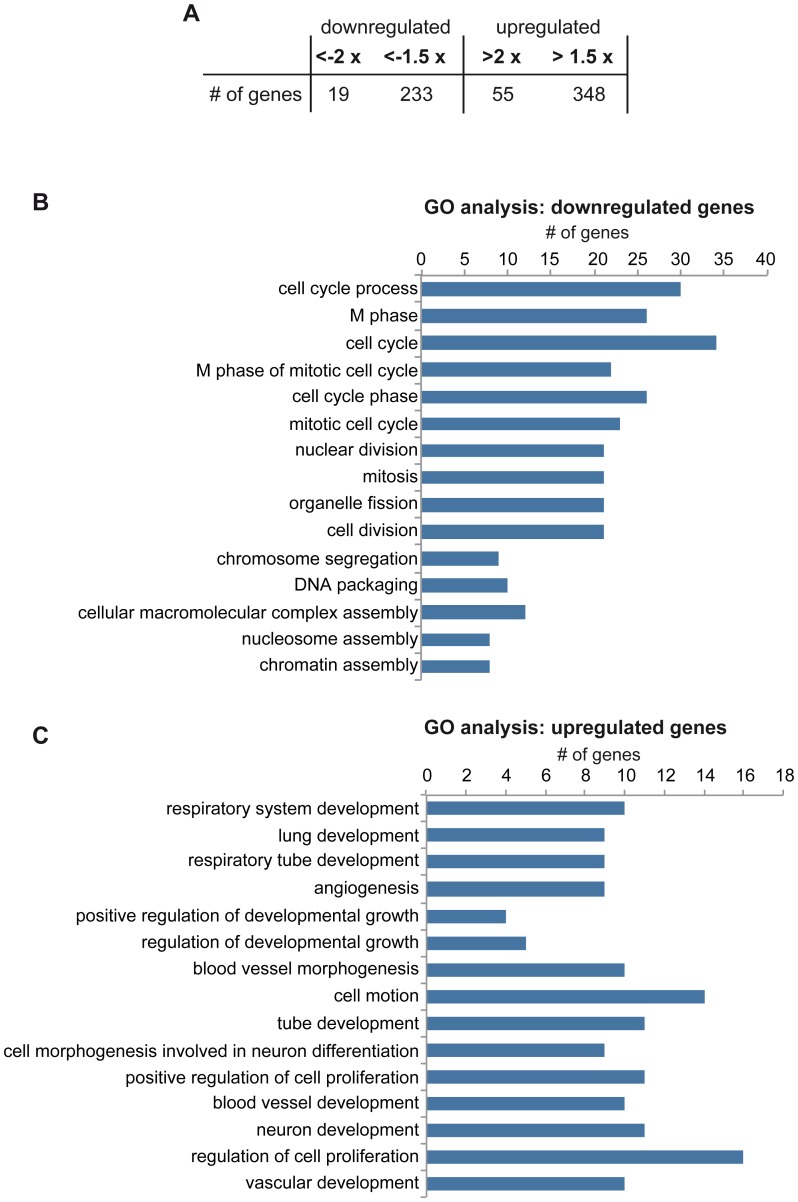
Gene expression changes after depletion of LIN9 in ESCs. (A) Number of up- and downregulated genes in LIN9 depleted cells identified by microarray analysis. For a list of regulated genes see Supplemental Table S1 (B) & (C) GO analysis was applied to differentially expressed genes. Listed are the top fifteen overrepresented GO terms according to the p-value. For complete lists of GO terms with a p-value of less than 0.05 see Supplemental Table S2 and S3.

To identify changes in gene expression that could be responsible for altered cell cycle progression after depletion of LIN9 in ESCs, we performed gene expression profiling using microarray analysis. RNA was isolated from control cells and from cells transfected with the LIN9 specific shRNA plasmid and subjected to Agilent DNA microarrays monitoring more than 39,000 transcripts. In total 581 genes showed >1.5 fold expression changes between control cells and LIN9 depleted cells (Fig. 4A, Supplemental Table S1). Of these, 348 genes were upregulated and 233 genes downregulated in LIN9 depleted cells compared to control cells (Fig. 4A).

To gain insights into the deregulated processes in ESCs after depletion of LIN9 we performed a gene ontology analysis (GO). This analysis indicated genes associated with cell cycle, mitosis and chromosome segregation were strongly overrepresented among the downregulated genes, consistent with the cell cycle phenotype of LIN9 depleted ESCs (Fig. 4B, Supplemental Table S2). GO terms overrepresented in the upregulated genes include lung development, angiogenesis, and neuronal development. This indicates that LIN9 directly or indirectly represses genes associated with differentiation and development (Fig. 4C, Supplemental Table S3).

**Figure 5:**
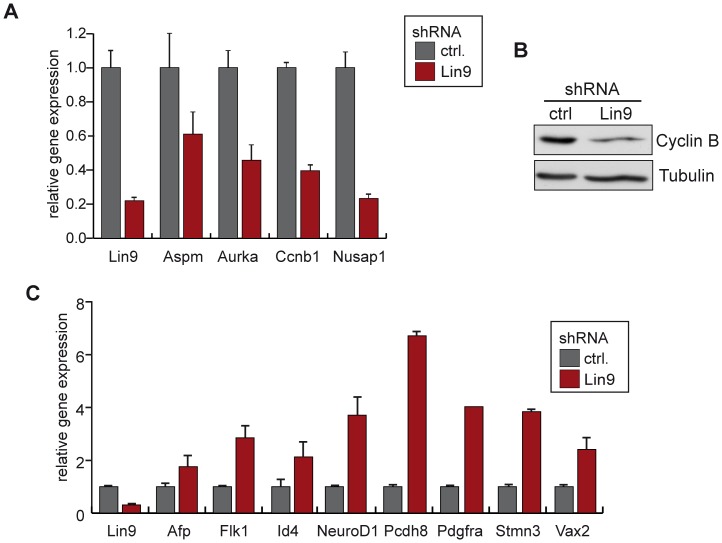
Validation of LIN9 target genes in ESCs. (A) & (C) Validation of microarray results by RT-qPCR. The expression of the indicated genes in control transfected cells and cells transfected with pSUPER-LIN9 was compared. (B) Expression of Cyclin B1 in control transfected ESCs and ESCs transfected with pSUPER-LIN9 was analyzed by immunoblotting. Tubulin was used as control for equal loading.

We validated the regulation of a subset genes discovered in the microarray by quantitative real time PCR (RT-qPCR) (Fig. 5A,C). These experiments confirmed downregulation of mitotic genes such as Aurora A, Cyclin B1 and Nusap1 (Fig. 5A). Decreased expression of mitotic Cyclin B was also confirmed on protein level by immunoblotting (Fig 5B). Upregulation of differentiation markers upon depletion of LIN9 including Id4, Vax2, Neurod1 and Stmn3 could also be confirmed (Fig. 5C).

### Genome Wide Binding of LIN9 to Promoters in ESCs

**Figure 6:**
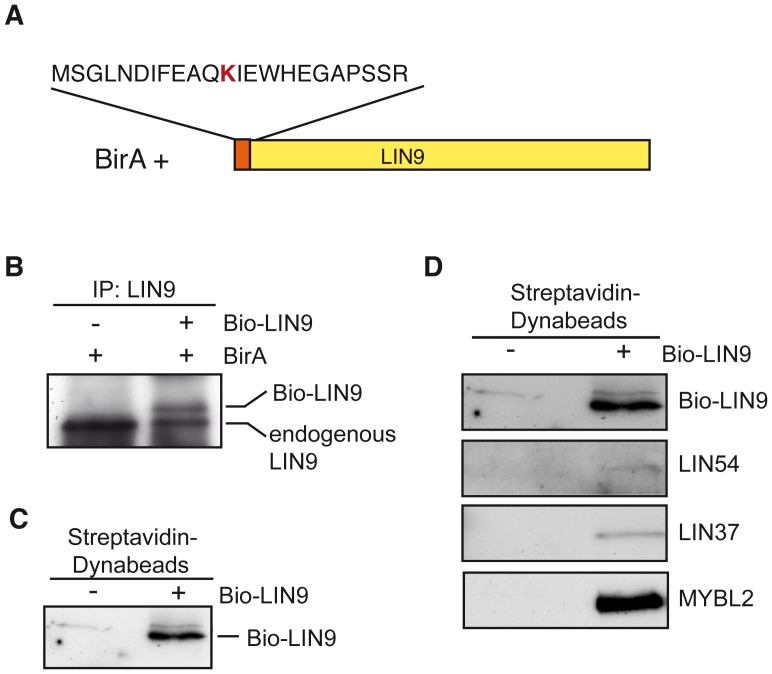
Generation of a Biotag-LIN9 ESC line. (A) Scheme of N-terminal tagged LIN9 with the BirA recognition sequence. The biotin acceptor lysine is indicated in red. BirA: E. coli biotin ligase (B) LIN9 was immunoprecipitated from ESCs stably expressing BirA alone or BirA and Biotag-LIN9. LIN9 was detected by immunoblotting. The positions of endogenous and Biotag-LIN9 are indicated. (C) LIN9 was affinity purified with streptavidin-coupled magnetic beads and detected by immunoblotting. (D) LIN9 was affinity purified with streptavidin-coupled magnetic beads. Bound proteins were detected by immunoblotting.

To determine which genes are direct transcriptional targets of LIN9 in ESCs, we performed a genome-wide ChIP-on-chip analysis of promoters bound by LIN9 in ESCs. To facilitate precipitation of LIN9 from ESCs, we first generated an ESC line stably expressing a tagged version of LIN9 with a recognition sequence for the biotinylating enzyme BirA, which mediates *in vivo* biotinylation of the tagged protein. A construct encoding for LIN9 fused to a biotin ligase recognition peptide (Bio-LIN9) was introduced into ESCs expressing BirA ligase [18] (Fig. 6A). Cells were selected with neomycin for 5 days. Single clones that express the LIN9 protein at similar level to endogenous LIN9 were identified by immunoblotting (data not shown and Fig. 6B). Pull-down assays using nuclear extract with streptavidin-coupled magnetic beads resulted in efficient and specific precipitation of LIN9 indicating that the tagged protein is efficiently biotinylated *in vivo* (Fig. 6C). Importantly, immunoblot analysis showed that B-MYB, LIN54 and LIN37 were present in streptavidin pulldown assays from Bio-LIN9 cells (Fig. 6D). This indicates that exogenous Bio-LIN9 is efficiently incorporated into the endogenous DREAM complex in ESCs.

**Figure 7:**
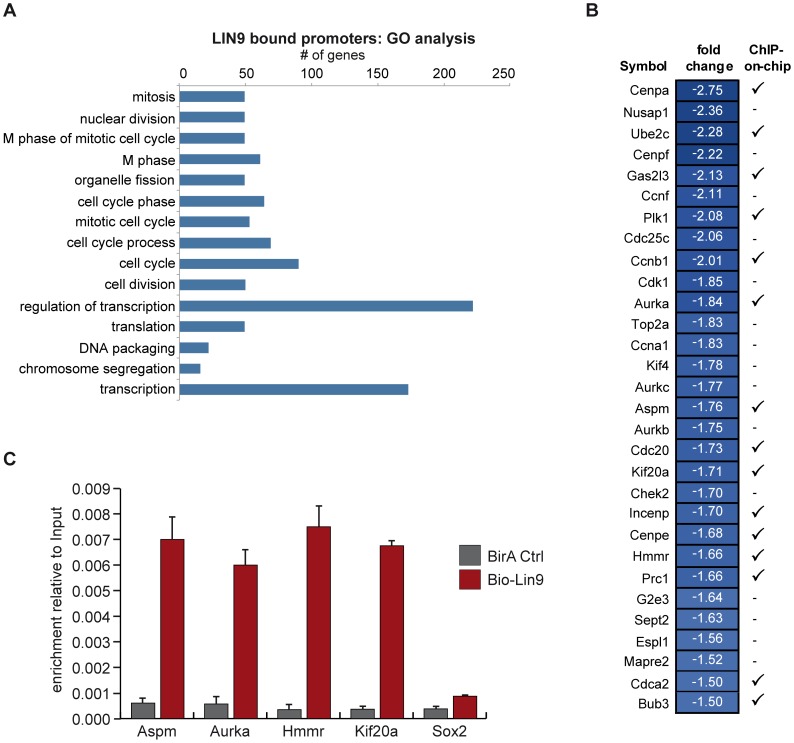
Identification of direct targets of LIN9 by ChIP-on-chip. (A) Functional categories of targets of LIN9 identified by ChIP-on-chip. LIN9 bound promoters were analyzed for enrichment of Gene Ontology terms. Shown are the top fifteen overrepresented GO terms according to the p-value. For a complete list bound promoters and GO terms with a p-value of less than 0.05 see Supplemental Tables S4 and S5. (B) Mitotic genes are direct targets of LIN9 in ESCs. Comparison of gene expression data and ChIP-on-chip data. Shown are genes that are downregulated after depletion of LIN9 and that have a known function in mitosis. “√” indicates that binding of LIN9 to the promoter was detected by ChIP-on-chip. “−” indicates that no binding was detected. (C) Binding of LIN9 to the promoters of randomly selected mitotic targets genes was confirmed by conventional ChIP.

To identify the promoters that were bound by LIN9 in ESCs, we next performed ChIP-on-chip experiments. Chromatin from cells expressing biotin-tagged LIN9 was precipitated with streptavidin coupled magnetic beads, amplified, labeled and hybridized to a microarray that contains oligonucleotide probes covering the region −2 to +0.5 kb relative to the transcriptional start sites of 19,489 annotated mouse genes. Overall, LIN9 bound to 1411 (7.2%) of the promoter regions analyzed (Supplemental S4). Functional annotation analysis based on GO terms revealed a significant enrichment on promoters of genes that are involved in regulation of mitosis, transcription, translation and mRNA processing (Fig. 7A and Supplemental Table S5).

By combining the ChIP-on-chip data with the microarray data, we determined which of the genes that change in expression upon depletion of LIN9 are directly regulated by LIN9.

Overall, only 59 of the 581 genes that that showed significant changes in gene expression had LIN9 bound in their promoter region (Table 1). 33 of the directly regulated genes were downregulated and 26 upregulated. Most significantly, LIN9 bound to the promoter regions of 15 of the 30 downregulated mitotic genes identified in the transcriptional profiling, indicating that the many of these genes are direct targets of LIN9 (Fig. 7B). Conventional chromatin immunoprecipitation (ChIP) experiments independently confirmed binding of LIN9 to the promoters of the mitotic genes Aspm, Aurora A, Hmmr and Kif20a in ESCs, while no association with the Sox2 promoter was detected (Fig. 7C). In contrast to the downregulated genes, the upregulated genes bound by LIN9 in ESCs cannot be assigned to distinct biological processes or pathways.

**Table 1:** Direct target genes of LIN9 in ES cells as determined by microarray and ChIP-on-chip.

Gene name	Direction	Gene Description
**2900011O08Rik**	down	RIKEN cDNA 2900011O08 gene
**3300002I08Rik**	up	RIKEN cDNA 3300002I08 gene
**4930547N16Rik**	down	RIKEN cDNA 4930547N16 gene
**A430089I19Rik**	up	RIKEN cDNA A430089I19 gene
**A530032D15Rik**	up	Sp140 nuclear body protein
**Acpp**	up	acid phosphatase, prostate
**Aspm**	down	asp (abnormal spindle)-like, microcephaly associated (Drosophila)
**Aurka**	down	aurora kinase A
**BC028528**	up	cDNA sequence BC028528
**Bub3**	down	budding uninhibited by benzimidazoles 3 homolog (S. cerevisiae)
**Ccnb1**	down	cyclin B1
**Cdc20**	down	cell division cycle 20 homolog (S. cerevisiae)
**Cdca2**	down	cell division cycle associated 2
**Cenpa**	down	centromere protein A
**Cenpe**	down	centromere protein E
**Cpne8**	up	copine VIII
**Ctss**	up	cathepsin S
**Cyp2d26**	down	cytochrome P450, family 2, subfamily d, polypeptide 26
**D17H6S56E-5**	down	DNA segment, Chr 17, human D6S56E 5
**Depdc1b**	down	DEP domain containing 1B
**Dst**	up	dystonin
**Fbp2**	up	fructose bisphosphatase 2
**Gas2l3**	down	growth arrest-specific 2 like 3
**Gm9**	down	predicted gene 9
**Grn**	down	granulin
**H1f0**	down	H1 histone family, member 0
**Hist1h2bc**	down	histone cluster 1, H2bc
**Hmga2**	up	high mobility group AT-hook 2
**Hmmr**	down	hyaluronan mediated motility receptor (RHAMM)
**Igfbp7**	up	insulin-like growth factor binding protein 7
**Incenp**	down	inner centromere protein
**Inhbc**	up	inhibin beta-C
**Kif20a**	down	kinesin family member 20A
**Lmnb1**	down	lamin B1
**Lrp4**	up	low density lipoprotein receptor-related protein 4
**Ly6a**	down	lymphocyte antigen 6 complex, locus A
**Matn3**	up	matrilin 3
**Msc**	down	musculin
**Oasl1**	up	2'–5' oligoadenylate synthetase-like 1
**Pipox**	up	pipecolic acid oxidase
**Plk1**	down	polo-like kinase 1 (Drosophila)
**Pnrc2**	down	proline-rich nuclear receptor coactivator 2
**Polr3gl**	up	polymerase (RNA) III (DNA directed) polypeptide G like
**Prc1**	down	protein regulator of cytokinesis 1
**Prom1**	up	prominin 1
**Prr11**	down	proline rich 11
**Psat1**	down	phosphoserine aminotransferase 1
**Rlbp1**	down	retinaldehyde binding protein 1
**Slc30a9**	up	solute carrier family 30 (zinc transporter), member 9
**Snap91**	up	synaptosomal-associated protein 91
**Socs2**	up	suppressor of cytokine signaling 2
**Speer4d**	up	spermatogenesis associated glutamate (E)-rich protein 4d
**Spnb1**	up	spectrin beta 1
**Syt10**	down	synaptotagmin X
**Tax1bp1**	up	Tax1 (human T cell leukemia virus type I) binding protein 1
**Tpm1**	up	tropomyosin 1, alpha
**Ube2c**	down	ubiquitin-conjugating enzyme E2C
**Zfp28**	down	zinc finger protein 28
**Zfp521**	up	zinc finger protein 521

As shown above, the depletion of LIN9 in ESCs resulted in upregulation of several differentiation specific genes such as Id4, NeuroD1, Vax2, Tbx4 and Socs2. However, binding of LIN9 to the promoters of these genes was not detected in the ChIP-on-chip analysis, suggesting that LIN9 indirectly regulates these genes.

B-MYB, which is also a part of the DREAM activating complex, has recently been shown to bind to the promoters of pluripotency genes Sox2, Nanog and Pou5f1 in ESCs [13], [14]. However, no binding of LIN9 to the promoters of these genes as well as to other pluripotency genes such as Klf4 was detected by ChIP-on-chip (Figure 7C, Supplemental Table S4). This result is consistent with unchanged expression of these genes upon depletion of LIN9 (see Fig. 3B).

In conclusion the ChIP-on-chip analysis demonstrated that mitotic genes are directly regulated by LIN9. In contrast, the expression of differentiation genes appears to be indirectly regulated by LIN9. Furthermore LIN9 does not bind to or regulate the expression of pluripotency genes.

## Discussion

We describe the first genome-wide analysis of genes regulated by the DREAM-subunit LIN9 in murine ESCs by using a combination of RNAi mediated depletion of LIN9 and microarray experiments. We found that LIN9 is required for the activation of key mitotic genes such as Plk1, Aurora A and Cyclin B in ESCs. Genome wide ChIP-on-chip experiments showed that mitotic genes are direct targets of LIN9. The consequences of depletion of LIN9 in ESCs are impaired proliferation and drastically reduced embryoid body formation, an *in vitro* model of differentiation. Furthermore, depletion of LIN9 resulted in accumulation of cells in G2/M and in polyploidy. These data indicate that LIN9 is required for proper maintenance of genome stability in ESCs because it functions as a master regulator of mitotic gene expression.

The role of LIN9 in regulating ESC proliferation is consistent with the results from our *in vivo* studies shown that deletion of LIN9 in mice leads to early embryonic lethality [11]. *Lin9* knockout embryos develop to the blastocyst stage and implant but die shortly thereafter because cell types that are derived from the inner cells mass (ICM) are not maintained after implantation [11]. In addition, in *in vitro* cultures of Lin9 mutant blastocysts the ICM is not maintained. The experiments described here suggest that the embryonic lethality of *Lin9−/−* mice is due to defects in ICM proliferation and due to genome instability because of a global reduction in mitotic gene expression. After implantation of the mouse embryo, epiblast cells form a single cell layer of columnar epithelium. Before gastrulation the epiblast undergoes a rapid cell proliferation, with cell cycles as short as 5 hours, to expand the pluripotent cell population from 25 cells at 4.5 dpc to ∼660 cells by 6.5 dpc [19]. It is likely that a failure of epiblast proliferation contributes to the lethality of Lin9 knockout embryos. Consistent with this notion several of the mitotic targets of LIN9 are also essential during early murine development. For example, inactivation of LIN9 targets Cyclin B1 or Plk1 results in embryonic lethality [20],[21]. In addition, LIN9 target genes Bub3, Cenpa, Cenpe and Incenp that function in mitotic checkpoint and kinetochore function, are also required for cell viability at the peri-implantation stage [22],[23],[24],[25]. This suggests that abnormal mitotic progression due to downregulation of these genes contributes to the lethal phenotype of Lin9 embryos.

LIN9 depletion in ESCs not only resulted in downregulation of mitotic genes but also in upregulation of lineage-specific differentiation markers such as NeuroD1, Flk1, Afp or Gata6. No binding of LIN9 to the promoters of these and other differentiation genes was observed, indicating that these differentiation-specific genes are upregulated indirectly. It is possible that their activation in the absence of LIN9 is mediated by activating E2F proteins. However, except for one gene (Pdgfra) they have not been described as E2F-targets before [26]. Thus, their upregulation could be an indirect consequence of changes in cell cycle progression. Despite upregulation of differentiation genes, AP staining was maintained and expression of pluripotency markers such as Oct4 and Sox2 was not decreased after depletion of LIN9. This indicates that the cells are undifferentiated and that they maintain their self-renewal capacity and pluripotency. Thus, although depletion of LIN9 results in a shift towards expression of markers of the differentiated state, it does not induce widespread differentiation and it does not result in loss of pluripotency. During lineage diversification, the upregulation of lineage specific genes is normally correlated with a downregulation of self-renewal genes. However, when LIN9 is depleted this process is disrupted and although lineage specific genes are induced, the expression of pluripotency genes is maintained.

The relationship between cell cycle regulation of ESCs and self-renewal is not well understood. Embryonic stem cells have a very short G1 phase and a high proliferation rate, which may be required to maintain their pluripotent state [27]–[31]. Specifically, it has been proposed that the short G1 phase minimizes their sensitivity to differentiation signals and thus helps to prevent ESCs from inappropriate differentiation. Thus, an extended G1 phase could make cells more susceptible to differentiation signals and may be a prerequisite of ESC differentiation. However, in a recent study it was shown that simply prolonging the G1 phase of mouse ESCs is not sufficient to induce their differentiation [4]. Whether normal progression through G2 and mitosis is functionally linked to pluripotency has not been investigated. We find that reduced proliferation due to a decreased expression of mitotic genes after depletion of LIN9 is not sufficient to induce differentiation. This supports the notion that simply lengthening the ESC cell cycle does not automatically promote ESC differentiation.

Our finding that LIN9 is required for expression of mitotic genes in ESCs is in agreement with the known function of LIN9 in regulation of mitotic genes in differentiated cells [9], [10]. In somatic cells, LIN9 is part of the conserved DREAM multiprotein complex that dynamically interacts with p130 and E2F4 early in quiescent cells or with B-MYB in S phase and G2 [7], [8]. Consistent with the recent description of a DREAM-like complex in embryonal carcinoma F9 cells [32], we find that LIN9 associates with the other core-subunits of DREAM and with B-MYB, but not with pocket proteins or E2F4 in ESCs. This suggests that LIN9 activates mitotic genes in ESCs in a complex together with DREAM and B-MYB. The lack of binding of DREAM to pocket proteins was expected, because pocket proteins are inactivated in ESCs by hyperphosphorylation due to high levels of cyclin E-cdk2 activity [3]. Because DREAM does not interact with pocket proteins in ESCs, these observations suggest that B-MYB/DREAM acts directly in transcriptional activation of G2/M genes as opposed to a possible indirect effect on inhibitory DREAM/E2F4-p130 complexes.

While B-MYB has recently implicated in maintaining the expression of pluripotency genes such as Oct4, Sox2 and Nanog in ESCs [13], [14], in another study using a conditional allele of B-MYB no change in expression of pluripotency genes after deletion of B-MYB was detected [33]. Because the levels of pluripotency genes are not changed after depletion of LIN9, our data do not support a regulation of pluripotency genes by DREAM. Whether B-MYB regulate these genes independently of DREAM remains to be investigated.

We note that only a small proportion of the LIN9 bound genes are differentially expressed in ESCs. It is possible that some of the bound genes are enriched non-specifically e.g. by non-specific amplification during the amplification step that is required before hybridization to the promoter array. It is also possible that changes in expression require a complete loss of LIN9 as opposed to the partial depletion by RNAi. Finally these targets may be regulated in different conditions, for example in another tissues or cell type. The specific combination of other transcriptional regulators that also contribute to the expression of these genes may determine which genes are regulated by LIN9 in a given context.

Altogether our data suggest that the LIN9 subunit of the DREAM complex is required for proliferation and genome stability of ESCs through the regulation of key mitotic genes.

## Materials and Methods

### ESC Culture

Mouse ESCs (E14) [34] were cultured in DMEM (Life Technologies) supplemented with 15% FCS (Life Technologies) 1000 u/ml LIF, non-essential amino acids, penicillin-streptomycin, 0.1 mM ß-mercapto-ethanol, 1 mM Na-pyruvate (Life Technologies) on gelatin-coated dishes. To generate an ESC line in which LIN9 is metabolically labeled by biotin, the Lin9 cDNA was cloned into the pEF-Flag-Bio plasmid [35] and the resistance gene was exchanged with a neomycin resistance gene. pEF-FLAG-Bio-Lin9 (neo) was transfected into ESCs that express the BirA enzyme from the ROSA26 locus [18]. After selection with neomycin (400 µg/ml), resistant clones were screened by immunoblotting and clones with equal levels of endogenous and biotinylated LIN9 were identified.

### Alkaline Phosphatase (AP) Activity

Alkaline Phosphatase (AP) activity was determined with the Alkaline Phosphatase Detection Kit (Millipore).

### In situ Hybridization

RNA in situ hybridization was performed as described previously except that glass needles where used in instead of a WISH chamber system [36].

### Embryoid Body Formation

For embryoid body formation the hanging-drop-method was used. To do so, an ESC suspension was prepared with 1×10^4^ cells/ml in LIF free ES medium. Drops of 20 µl (200 cells) were placed on the lid of cell culture dishes and carefully placed on a dish with 1×PBS to prevent the cells from drying out. After 2 days incubation, EBs were harvested and cultured in suspension on dishes coated with 20 mg/ml poly(2-hydroxymethyl methacrylate) (Sigma).

### Immunoprecipitation and Immunoblotting

Nuclear extracts were prepared as described before [37]. Briefly cells were lysed with Nuclear Extraction Buffer A [20 mM HEPES, 10 mM KCl, 1 mM EDTA, 0.1 mM Na3VO4, 0.2% (v/v) Nonidet P40 (NP-40), 10% (v/v) glycerol, 1 mM DTT, 1 mM PMSF, protease inhibitor cocktail (Sigma)]. Nuclei were collected by centrifugation and nuclear proteins were extracted with Nuclear Extraction Buffer B [20 mM HEPES, 10 mM KCl, 1 mM EDTA, 0.1 mM Na3VO4, 350 mM NaCl, 20% (v/v) glycerol, 1 mM DTT, 1 mM PMSF, protease inhibitor cocktail (Sigma)]. Nuclear extracts were immunoprecipitated with LIN9 antibody (ab62329, Abcam) over night at 4°C, bound to Protein-G Dynabeads (Life Technologies) and washed 5 times with TNN [50 mM Tris (pH 7.5), 120 mM NaCl, 5 mM EDTA, 0.5% NP40, 10 mM Na_4_P_2_O_7_, 2 mM Na_3_ VO_4_, 100 mM NaF, 10 mg/mL phenylmethylsulfonyl fluoride, protease inhibitors (Sigma)]. Proteins were separated by SDS-PAGE, transferred to PVDF membrane and detected by immunoblotting. The following primary antibodies were used: p107 (sc-318), p130 (sc-317), CCNB1 (sc-245) (all from Santa Cruz) and Tubulin (B-5-1, Sigma). The LIN9, LIN54, and LIN37 antibodies have been described before [7], The MYBL2 antibody (LX015.1) has also been described [38].

### RNAi

pSUPER-puro Lin9 was generated by inserting an oligonucleotide with the following target sequence specific for mouse Lin9 into pSUPER-puro: 5' GCUACUUACAGAGUAACUUUC 3' [32]. ESCs were transfected with Lipofectamine 2000 (Invitrogen) and selected with 1 mg/ml puromycin (Invivogen).

### Chromatin-Immunoprecipitation (ChIP) and ChIP-on-chip

For ChIP, 5×10^7^ cells were cross-linked with 1% formaldehyde for 10 min at room temperature. Cross-linking was terminated by 125 mM glycine and cells were washed with cold phosphate-buffered saline (PBS) containing 10 mg/mL phenylmethylsulfonyl fluoride, collected by centrifugation and washed again. The cell pellet was resuspended in SDS ChIP buffer [0.1% SDS, 1% Triton X-100, 2 mM EDTA, 20 mM Tris-Cl pH 8.1, 150 mM NaCl, and protease inhibitor cocktail (Sigma)]. DNA was fragmented to an average size 0.5–1 kb with the Bioruptor sonificator from Diagenode. Chromatin was precipitated by addition of BSA and ssDNA pre-blocked Streptavidin M250 Dynabeads (Life Technologies) and incubated for 5 hours. Precipitated chromatin was successively washed with buffer I (2% SDS), buffer II [0.1% Deoxycholate, 1% Triton X-100, 1 mM EDTA, 50 mM HEPES pH 7.5, 500 mM NaCl], buffer III [250 mM LiCl, 0.5% NP-40, 0.5% Deoxycholate, 1 mM EDTA, 10 mM Tris-Cl pH 8.1] and TE buffer [10 mM Tris-Cl pH 7.5, 1 mM EDTA]. SDS elution buffer [50 mM Tris-Cl pH 8.0, 1% SDS, 10 mM EDTA] was added and incubated at 65°C overnight to reverse the crosslink. The sample was treated with 1 µl RNase A [10 mg/ml] and 2 µl Proteinase K [10 mg/ml]. DNA was purified with the Qiagen PCR Purification Kit according to the manufacture’s protocol and resuspended in 25 µl H_2_O. Enrichment of DNA was analyzed by quantitative real-time PCR. Primer sequences are listed below.

For ChIP-on-chip, crosslinked chromatin was prepared from 2×10^8^ Bio-Lin9 cells and precipitated with streptavidin-coupled Dynabeads (Life Technologies) as described above. The crosslink was reversed, the DNA eluted and purified. Precipitated samples and input DNA were amplified using the WGA2 kit (Sigma). 4 µg amplified DNA was labelled with Cy5 and Cy3 and hybridized to a MM8 385 k NimbleGen Mouse ChIP 385 k RefSeq promoter array containing oligonucleotide probes that cover the region −2 to +0.5 kb relative to the transcriptional start sites for 19,489 annotated transcripts. Probes consisted of 50- to 75-mers at approximately 100 bp spacing. DNA labeling, hybridization, detection and data analysis were performed using the services of Imagenes (Berlin). Signal intensity data were extracted from the scanned images of each array using NimbleScan data extraction software. Log_2_ ratio of experimental and input signals was then computed and scaled and peak data files were generated (.gff) by identifying four or more consecutive probes, whose signals are above a cutoff value (a percentage of the hypothetical maximum (mean +6 standard deviation) using a 500 bp sliding window. The probability of false discovery is calculated by randomizing the data 20 times and each peak is given a false discovery rate (FDR), where the lower the FDR score, the higher the probability that peak represents a true binding site. Enriched peaks were visualized using SignalMap. Promoters with a FDR <0.1 and a peak score >1 were considered positive LIN9 targets.

### Microarray

Using the two color Quick-Amp Labeling Kit (Agilent) 0.1 µg of total RNA was used for cDNA synthesis, mRNA amplification and labeling according to manufacturer’s instructions. Transcriptional profiling was done on an 44 K oligo array (Agilent) and analyzed as described before [39]. Expression data and gene annotations were stored in Array Express (http://www.ebi.ac.uk/arrayexpress/) (accession: E-MTAB-1490), which complies with MIAME (minimal information about a microarray experiment) guidelines.

### Real-time Quantitative PCR

Total RNA was isolated with Trifast (Peqlab), reverse transcribed with 0.5 units M-MLV-RT Transcriptase (Thermo Scientific) and analyzed with quantitative real-time PCR with SYBR Green reagents from Thermo Scientific using the Mx3000 (Agilent technologies) detection system. Expression differences were calculated relative to GAPDH as described before [7].

### FACS Analysis

For FACS analysis cells were stained with propidium iodide and analyzed on a Beckman Coulter FC500.

### Primer Sequences

Primers for RT-qPCR:

β-actin

5′-CTAAGGCCAACCGTGAAAAG-3′ forward

5′-ACCAGAGGCATACAGGGACA-3′ reverse

Lin9

5′-TTGGGACTCACACCATTCCT-3′ forward

5′-GAAGGCCGCTGTTTTTGTC-3′ reverse

Ccnb1

5′-CGCTGAAAATTCTTGACAACG-3′ forward

5′-TCTTAGCCAGGTGCTGCATA-3′ reverse

Aspm

5′-GATGGAGGCCGAGAGAGG-3′ forward

5′-CAGCTTCCACTTTGGATAAGTATTTC-3′ reverse

Nusap1

5′-TCTAAACTTGGGAACAATAAAAGGA-3′ forward

5′-TGGATTCCATTTTCTTAAAACGA-3′ reverse

Aurka

5′-GGGACATGGCTGTTGAGG-3′ forward

5′-GTTTTCTTTACATCTGTCCATGTCA-3′ reverse

Oct4

5′-GTTGGAGAAGGTGGAACCAA-3′ forward

5′-CTCCTTCTGCAGGGCTTTC-3′ reverse

Flk1

5′-CAGTGGTACTGGCAGCTAGAAG-3′ forward

5′-ACAAGCATACGGGCTTGTTT-3′ reverse

Vax2

5′-ACTGAGTTGGCCCGACAG-3′ forward

5′-CCCGCTTCTCCAGGTCTC-3′ reverse

NeuroD1

5′-CGCAGAAGGCAAGGTGTC-3′ forward

5′-TTTGGTCATGTTTCCACTTCC-3′ reverse

Gm11487

5′-AGCTCAGGAGACAAAATGCAG-3′ forward

5′-CTGAGGAACTTTGGCCTTCTT-3′ reverse

AFP

5′-CATGCTGCAAAGCTGACAA-3′ forward

5′-CTTTGCAATGGATGCTCTCTT-3′ reverse

Pcdh8

5′-GAAGTTCAGTGGGAAAGACAGC-3′ forward

5′-GTACACGCCCACAGTCCAC-3′ reverse

Pdgfra

5′-GTCGTTGACCTGCAGTGGA-3′ forward

5′-GTCGTTGACCTGCAGTGGA-3′ reverse

Stmn3

5′-CTGAGGAGCGGAGGAAGA-3′ forward

5′-CCTCCCGTTCATGCTCAC-3′ reverse

Id4

5′-AGGGTGACAGCATTCTCTGC-3′ forward

5′-CCGGTGGCTTGTTTCTCTTA-3′ reverse

Primers for ChIP:

Kif20a

5′-CAGACAGTCTTCGGGTGAGTG-3′ forward

5′-CTTCTACGGACGCGCAAG-3′ reverse

Hmmr

5′-TCGCCTGAATTCAAATTTACC-3′ forward

5′-CAGGATTGGCCAGATAGGTT-3′ reverse

Aurka

5′-AGAAGGCTGCGGGAAGAG-3′ forward

5′-GTCTGTGGATGAACGGGAGT-3′ reverse

Aspm

5′-GCTGTAGCGAGGAGGTTCC-3′ forward

5′-TTTTGCTCGGTTCAAATATCG-3′ reverse

Sox2

## Supporting Information

Figure S1 **Embryoid bodies formed in control cells and LIN9-depleted cells.** Scale bar: 100 µM. See also Figure 2D.
(PDF)

Table S1 **LIN9 regulated genes in ESCs as determined by microarray analysis.**
(XLS)

Table S2 **GO term analysis of genes downregulated after depletion of LIN9.**
(XLSX)

Table S3 **GO term analysis of genes upregulated after depletion of LIN9.**
(XLSX)

Table S4 **Genes bound by LIN9 based on CHIP-on-chip analysis.**
(XLSX)

Table S5 **GO term analysis of genes bound by LIN9.**
(XLSX)
