# Supplementary figures and images for: LIN9, a Subunit of the DREAM Complex, Regulates Mitotic Gene Expression and Proliferation of Embryonic Stem Cells
Source: PLoS One. 2013 May 7;8(5):e62882. doi: 10.1371/journal.pone.0062882 (PMC3647048; doi:10.1371/journal.pone.0062882)

Supplementary Figure S1

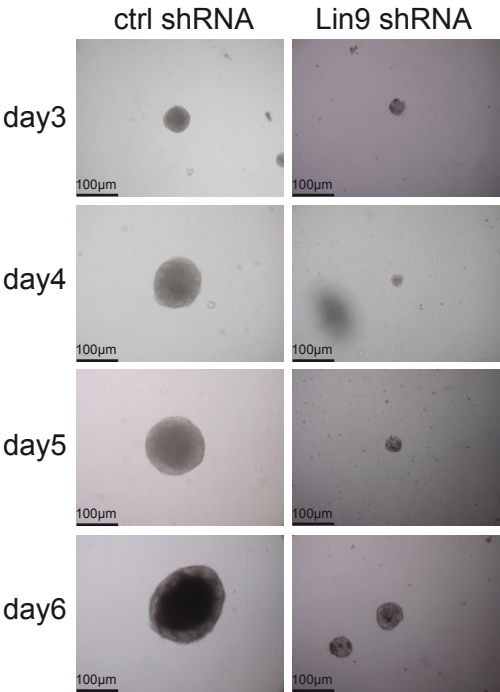

Supplement: Figure S1 — Embryoid bodies formed in control cells and LIN9-depleted cells. Scale bar: 100 µM. See also Figure 2D. (PDF) [file pone.0062882.s001.pdf]
